# Vaccination of Sheep with a Methanogen Protein Provides Insight into Levels of Antibody in Saliva Needed to Target Ruminal Methanogens

**DOI:** 10.1371/journal.pone.0159861

**Published:** 2016-07-29

**Authors:** Supatsak Subharat, Dairu Shu, Tao Zheng, Bryce M. Buddle, Kan Kaneko, Sarah Hook, Peter H. Janssen, D. Neil Wedlock

**Affiliations:** 1 AgResearch, Hopkirk Research Institute, Grasslands Research Centre, Palmerston North, New Zealand; 2 School of Pharmacy, University of Otago, Dunedin, New Zealand; Federal University of Pelotas, BRAZIL

## Abstract

Methane is produced in the rumen of ruminant livestock by methanogens and is a major contributor to agricultural greenhouse gases. Vaccination against ruminal methanogens could reduce methane emissions by inducing antibodies in saliva which enter the rumen and impair ability of methanogens to produce methane. Presently, it is not known if vaccination can induce sufficient amounts of antibody in the saliva to target methanogen populations in the rumen and little is known about how long antibody in the rumen remains active. In the current study, sheep were vaccinated twice at a 3-week interval with a model methanogen antigen, recombinant glycosyl transferase protein (rGT2) formulated with one of four adjuvants: saponin, Montanide ISA61, a chitosan thermogel, or a lipid nanoparticle/cationic liposome adjuvant (n = 6/formulation). A control group of sheep (n = 6) was not vaccinated. The highest antigen-specific IgA and IgG responses in both saliva and serum were observed with Montanide ISA61, which promoted levels of salivary antibodies that were five-fold higher than the second most potent adjuvant, saponin. A rGT2-specific IgG standard was used to determine the level of rGT2-specific IgG in serum and saliva. Vaccination with GT2/Montanide ISA61 produced a peak antibody concentration of 7 × 10^16^ molecules of antigen-specific IgG per litre of saliva, and it was estimated that in the rumen there would be more than 10^4^ molecules of antigen-specific IgG for each methanogen cell. Both IgG and IgA in saliva were shown to be relatively stable in the rumen. Salivary antibody exposed for 1–2 hours to an *in vitro* simulated rumen environment retained approximately 50% of antigen-binding activity. Collectively, the results from measuring antibody levels and stablility suggest a vaccination-based mitigation strategy for livestock generated methane is in theory feasible.

## Introduction

Vaccination against rumen methanogens has the potential to reduce methane emissions from livestock, which is a major contributor to agricultural greenhouse gases [1–3]. The concept is to induce salivary anti-methanogen antibodies which are delivered to the rumen and reduce the activity of methane-producing methanogens. Vaccinating sheep and cattle against the rumen dwelling organisms *Streptococcus bovis* and *Lactobacillus* species, the major etiological microbes responsible for acute ruminal acidosis, has shown that antibodies can translocate to the rumen via saliva and protect against lactic acidosis [4–9]. A recent study in cattle has shown that vaccination against the alpha subunit of *Helicobacter pylori* urease can reduce ureolytic activity in the rumen [10].

In addition to selecting optimal antigens, an effective anti-methanogen vaccine will need to induce sufficiently high levels of salivary antibodies to bind to specific targets on the rumen methanogens [11]. To date, little is known about the levels and type of antibody that need to be generated in the saliva and delivered to the rumen and also whether these antibodies persist long enough within the rumen environment for a vaccine to be effective. The first aim of the current study were to determine the levels of the major class (IgG and IgA) of immunoglobulin (Ig) in saliva and the rumen of sheep and determine how long antibodies can retain their activity in the rumen. A second aim was to identify a suitable adjuvant that will result in high levels of anti-methanogen antibodies in the saliva. A vaccine trial was conducted in sheep using a previously identified methanogen protein, glycosyl transferase (GT2) [11] as a model antigen, and comparing different adjuvants. A chitosan gel designed for slow and sustained release of antigens [12–14] and cationic liposomes that target negatively charged cell membranes [15] were compared with two commercially available adjuvants, Montanide ISA61 and saponin. A third aim of the study was to provide an estimation of the number of antigen-specific antibody molecules produced in saliva following vaccination. An understanding of salivary antibody concentration will provide theroretical estimation into whether the current vaccination strategy produce enough antibody in the rumen to have an impact on methanogen activity.

## Materials and Methods

### Animals

Thirty 6-month-old female Romney lambs were used in the vaccine trial. The animals were sourced from a commercial sheep farm in the lower North Island of New Zealand. All animals were grazed on pasture with water *ad libitum* and monitored weekly for normal appearance and behaviour. None of the animals died during the experiment. At the end of the experiment, the animals were humanely euthanized in accordance with the New Zealand Ministry for Primary Industries code of welfare (sheep and beef cattle) 2010. This was carried out by stunning using a captive bolt and bleeding out. Animal ethics approval was obtained by the AgResearch Grassland’s Animal Ethics Committee, Palmerston North, New Zealand for all procedures involving animals.

### Preparation of vaccine

Recombinant GT2 (rGT2) was produced as the large extracellular domain (animo acids 23–247) from *Methanobrevibacter ruminantium* M1 (mru_2175) predicted using ConPred II. The DNA coding for the extracellular domain was synthesized (GeneArt; Life Technologies, USA) using *Escherichia coli* codon preference and subcloned into pET-32a (Novagen, USA) to create an inframe fusion protein with thioredoxin. The resultant construct was transformed into *E*. *coli* BL21 cells for production of recombinant protein using methods previously reported [16]. Briefly, the cells were harvested from the culture by centrifugation at 3,200 × g for 15 min at 4°C. The cell pellets were washed in ice cool NPI buffer (50 mM NaH_2_PO_4,_ 300 mM NaCl, pH 8.0 containing 10 mM imidazole (NPI-10)). Following recovery of the cells after centrigution at 3,200 × g for 10 min at 4°C, the cell pellet was re-suspended in ice cool NPI-10 at a ratio of 1 g of wet cell pellet per 3 mL NPI-10. Two milligrams of lysozyme and 10 μg of DNAse I were added and the mixture was incubated on ice for 30 min. The cells were disrupted by sonication using 10 × 20 s bursts with a 20 s cooling period between each burst. The crude lysate was centrifuged at 10,000 × g for 30 min at 4°C to remove cell debris and the supernatant filtered through a 0.45 μm membrane. The polyhistidine-tagged recombinant GT2 protein was purified by FPLC™ under native conditions using Protino® Ni-NTA Column (Macherey-Nagel, Germany) and a BIO-RAD chromatography system (Bio-Rad, USA). NPI-10 was used as the equilibration buffer, NPI containing 20 mM imidazole as the wash buffer and NPI containing 250 mM imidazole as the elution buffer. Following elution of protein, the imidazole was removed by dialysis against phosphate buffered saline (PBS) and the purified protein was stored at −20°C. SDS-PAGE analysis showed a protein band with the expected molecular weight of 42.2 kDa.

The rGT2 was formulated with the various adjuvants (Table 1). Each dose of vaccine for parenteral administration contained 0.1 mg rGT2 protein. Saponin (Sigma Chemicals, USA) was used at 5 mg per dose and Montanide ISA61 (Seppic, France) was used at a ratio of 6:4 w:w adjuvant:antigen. A sustained release chitosan thermogelling polymer formulation was produced by dissolving 1% chitosan and 0.5% methyl-cellulose in 0.05 M HCl. A 9% w/v solution of glycerol-2-phosphate disodium hydrate containing Quil A and rGT2 was slowly added to prepare the final vaccine containing 0.5 mg Quil A and 0.1 mg rGT2 per dose. Pegylated cationic Quil A and rGT2-loaded liposomes (9:1:1:2.2 molar ratio of phosphatidylcholine; 1,2-dioleoyl-3-trimethylammonium-propane; 1,2-dioleoyl-*sn*-glycero-3-phosphoethanolamine; 1,2-distearoyl-*sn*-glycero-3-phosphoethanolamine-*N*-(amino(polyethylylene glycol)-20000) (Avanti Polar Lipids, Inc., USA) were produced using the film hydration method [17].

**Table 1:** Vaccine groups, formulations and route of vaccination.

Animal group	Sample size (n)	rGT2	Adjuvant	Route of vaccination
1	6	0.1mg	Saponin	IM
2	6	0.1mg	Montanide ISA61	SC
3	6	0.1mg	Chitosan thermogel	SC
4	6	0.1mg	Lipid nanoparticles/ cationic liposomes	SC
5	6	−	−	−

SC, subcutaneous; IM, intramuscular

### Vaccination and sampling of animals

Thirty sheep were allocated randomly to 5 groups (Table 1). Animals in one group were given rGT2/ saponin by the intramuscular route (n = 6) while rGT2 formulated with Montanide ISA61 (n = 6), chitosan thermogel (n = 6) or lipid nanoparticles/cationic liposomes (n = 6) were administered subcutaneously to three other groups. A control group (n = 6) were not vaccinated. Animals were re-vaccinated at 3 weeks with the same vaccine. Vaccines were administered as a 2-mL dose in the anterior region of the neck. Blood samples were collected by jugular venepuncture, with serum separated and stored at −20°C. Saliva samples were collected using a cotton swab placed in mouth. The swab was then placed in a salivette saliva collection tube (Sarstedt, Germany), centrifuged at 2000 × g for 10 min, and the flow-through harvested and stored at −20°C. Rumen content samples were collected using a stomach tube, centrifuged at 10,000 × g for 15 min and the supernatants used for measurement of antibody. Blood and saliva samples were collected at weeks 0, 3, 6, 9 and 12 and rumen content samples collected at weeks 0, 6 and 12. Week 0 samples (n = 30) were used to determine level of total sheep Ig in serum, saliva and rumen content samples. Animals from the Montanide ISA61 adjuvant group were re-vaccinated at week 19 and blood and saliva sampled at weeks 19 and 21 to determine antibody longevity and effect of boosting. Control animals were sampled at week 19 and 21 for comparison.

### Determination of immunoglobulins stability in rumen contents

Fifteen paired sheep saliva and rumen content samples were used from vaccine trial at week 0 (n = 6, not vaccinated), week 6 (n = 6, all vaccinated) and week 12 (n = 3, all vaccinated) for determining stability of total Ig. For determining stability of rGT2-specific Ig, 10 paired sheep saliva and rumen content samples were used from vaccinated animals at week 12. Saliva from each animal was spiked into fresh rumen contents collected from the same animal at a 1:20 ratio estimated from published data on the rate of saliva inflow and rumen volume [18, 19] with or without protease inhibitor (cØmplete; Roche, Germany; 1 tablet/40 mL volume). Saliva was added to PBS as a control. The mixtures were incubated at 39°C (sheep body and rumen temperature [20]) for 0, 1, 2 or 4 h, placed on ice and then centrifuged (10,000 × g, 15 min, 4°C). Supernatants were removed and stored at 4°C until analysis for total Ig and rGT2-specific Ig by ELISA within 24hours (see below). Western blot analysis was performed to confirm the results obtained from ELISA. Sheep IgA purified from saliva, and sheep IgG (ChromPure; Jackson ImmunoResearch Laboratories, Inc., USA) (2.5 mg/mL in PBS) were spiked into rumen content samples at a 1:10 ratio with or without the protease inhibitor cocktail and incubated at 39°C for up to 4 h. Following centrifugation at 10,000 × g for 15 min, supernatants were subjected to SDS-PAGE followed by Western blot. Immunoglobulins were transferred onto a polyvinyl diflluoride membrane, followed by immunoblotting with rabbit anti-sheep IgA, horseradish peroxidase (HRP) conjugated (AbD Serotec, UK) and rabbit anti sheep IgG (H+L) HRP (Jackson ImmunoResearch).

### Measurement of antibodies using ELISA

#### Total sheep IgA and IgG ELISA.

ELISA plates (Nunc™; Thermo Fisher Scientific, Denmark) were coated overnight at 4°C with rabbit anti-sheep IgA (AbD Serotec) for total IgA or donkey anti-sheep IgG (AbD Serotec) for total IgG at a protein concentration of 1 μg/mL in PBS. The plates were blocked 2 h at room temperature with 150 μL/well of normal rabbit serum (0.1% v/v) in PBS for total IgA or combination of normal rabbit serum (0.5% v/v) and normal donkey serum (0.5% v/v) in PBS for total IgG. Serial dilutions of saliva (range 1:10^2^–1:10^5^), serum (range 1:10^3^–1:10^7^), rumen contents (range 1:10) or standards in PBS were added (100 μL/well) to wells in duplicate and plates were incubated for 2 h at room temperature. The plates were washed three times in PBS containing 0.1% Tween 20 (PBST) and incubated for 1 h at room temperature with 100 μL/well of either HRP-conjugated rabbit anti-sheep IgA (AbD Serotec) diluted 1:10,000 or HRP-conjugated rabbit anti-sheep IgG (Abcam, UK) diluted 1:10,000 in blocking buffer for IgA and IgG determination, respectively. The plates were washed three times in PBST and incubated for 10 min at room temperature in the dark with 100 μL of tetramethyl benzidine (TMB) substrate per well (BD OptEIA™; BD Biosciences, USA). The reactions were stopped with addition of 50 μL of 0.05 M H_2_SO_4_ per well and the absorbance read at 450 nm. A linear standard curve was fitted to the 2-fold serial dilution of the standards (100 ng/mL to 1.56 ng/mL) and used for the calculation of antibody concentration (μg/mL). Sheep IgA standard was obtained from purification of sheep saliva using anion-exhange chromatography followed by size exclusion gel filtration as described below whereas sheep IgG standard was obtained commercially (ChromPure; Jackson ImmunoResearch).

#### Antigen-specific IgA and IgG ELISA.

ELISA assays were developed to monitor rGT2-specific IgA and rGT2-specific IgG responses in serum, saliva and rumen contents. ELISA plates (Nunc™; Thermo Fisher Scientific) were coated overnight with 50 μL of rGT2 (4 μg/mL) per well in PBS at 4°C. The plates were blocked (1 h, room temperature) with 150 μL of 1% (w/v) casein in PBS per well. Serial dilutions of saliva (1:10–1:10^4^), serum (range 1:10–1:10^5^), rumen contents (only at 1:2) or standards in PBS were added (100 μL/well) to each of duplicate wells and plates incubated for 2 h at room temperature. The plates were washed three times in PBST and incubated for 1 h at room temperature with 100 μL/well of either HRP-conjugated rabbit anti-sheep IgA (AbD Serotec) or HRP-conjugated rabbit anti-sheep IgG (Abcam) diluted 1:5,000 in blocking buffer for rGT2-IgA and rGT2-IgG determination, respectively. The plates were washed three times in PBST and incubated for 10 min at room temperature in the dark with 100 μL of TMB substrate per well. The reactions were stopped with addition of 50 μL of 0.05 M H_2_SO_4_ per well and the absorbance read at 450nm. rGT2-specific IgA and IgG levels of the unknown samples were assigned a unit value based on the positive standard saliva and serum control, respectively. A linear standard curve was fitted to the 2-fold serial dilution of the positive saliva control (1:10 to 1:640) and positive serum control (1:100,000 to 1:6,400,000) and then used for the calculation of antibody concentration (units/mL) on every ELISA plate. Based on the standard curves, the rGT2-specific IgA concentration in the standard saliva (1:10 dilution) was assigned a value of 640 units/mL, while the rGT2-specific IgG concentration in the standard serum (1:100,000 dilution) was assigned a value of 6,400,000 units/mL. For saliva samples, rGT2-specific IgA and rGT2-specific IgG were standardised by total IgA or total IgG to control for animal variation in saliva flow rates. Saliva with visual blood contamination was excluded from analysis to prevent false positive antibody responses.

### Calculation of number of antigen-specific IgG molecules in sera and saliva

A rGT2-specific IgG standard was prepared as described below and used to determine the percentage of rGT2-specific IgG in serum and saliva by ELISA. A linear standard curve was fitted to the 2-fold serial dilution of the standards (100 ng/mL to 1.56 ng/mL) and used for the calculation of rGT2-specific IgG concentration (μg/mL). Sera and saliva taken at week 0, 3, 6, 9, 12, 19 and 21 from 6 animals vaccinated with rGT2/Montanide ISA61 group and 6 control animals were used. Antigen-specific IgG was expressed as a percentage of the total IgG in sera and saliva. The number of antigen-specific IgG molecules generated in saliva was calculated using the molecular weight of 150 kDa for IgG (2.49 g × 10^−19^ per molecule).

### Purification of sheep IgA standard

Saliva (30 to 50 mL) was collected from individual animal using a suction tube and pooled for further processing. Ethylene diamine tetra-acetate (EDTA, Sigma) and NaN_3_ (BDH, England) were added to saliva samples to a final concentration of 2 mM and 0.02% (w/v) respectively. The resultant solution was clarified by centrifugation at 15,000 × g, 4°C for 15 min. The mucus on the top and debris in the bottom were removed after centrifugation. Ammonium sulphate (Merck, Germany) was added slowly to the clarified saliva samples with gently stirring to a final concentration of 29% (w/v). Following incubation at 4°C for 16 h, the precipitate was recovered by centrifugation at 15,000 × g, 4°C for 1 h. The precipitated protein was dissolved in DEAE buffer (50 mM Tris-Cl, pH adjusted to 8.0 with HCl, and containing 0.02% (w/v) NaN_3_)_,_ followed by extensive dialysis in Cellu• Sep® T4 tubular dialysis membranes, molecular weight cut off 12 kDa (Interchim, France) against the same buffer to remove the ammonium sulphate. Purification of salivary IgA was performed in two steps: anion-exchange chromatography followed by gel filtration on Sephacryl S-300 using a BioLogic dual flow system and BioLogic DuoFlow software Version 5.0 (Bio-Rad Laboratories, Inc., USA). The crude IgA from the ammonium sulphate precipitation step was further diluted in DEAE buffer to at least two volumes of original sample volume before filtered through a 0.45 μm filter. The sample was loaded into Dynaloop 90 (Bio-Rad Laboratories, Inc.). The sample was auto-injected at a flow rate of 3 mL/min onto HiTrap DEAE sepharose fast flow 5 mL column (GE Healthcare, Sweden). Weakly bound proteins were washed off with DEAE buffer. Bound IgA was eluted using DEAE buffer with a shallow linear gradient from 0.2 to 0.4 M NaCl at a flow rate of 2 mL/min. Eluted IgA was collected in 2-mL fractions, and the total eluted fraction containing IgA were subsequently concentrated down to less than 5 mL prior to enrichment of IgA using size-exclusion chromatography. Sephacryl S-300 high resolution HiPrep 26/60 (GE Healthcare), was equilibrated with Tris buffer saline (50 mM Tris-Cl, pH adjusted to 8.0 with HCl, 150 mM NaCl containing 0.02% (w/v) NaN_3_)_,_ the concentrated elution was loaded and 2.5 mL fractions were collected at a flow rate of 1.3 mL/min. Fractions containing purified IgA were combined and concentrated and the purified IgA was analysed by Western blotting and ELISA. No contaminating IgG was detected in the IgA by either ELISA or Western blotting.

### Purification of antigen-specific sheep IgG

A HiTrap NHS-activated column (GE HeathCare) was used for the enrichment of GT2-specific IgG from anti-sera generated against rGT2. Recombinant GT2 was coupled onto a 1-mL HiTrap NHS-activated column) as follows. Briefly, rGT2 (2 mL at 8.2 mg/mL) in coupling buffer (0.2 M NaHCO_3_, 0.5 M NaCl, pH 8.3). was loaded onto the column at a flow rate of 0.3 mL/min. The column was sealed and kept at room termperature for 30 minutes after a 1 mL injection. Excess ligand was removed with coupling buffer at a flow rate of 1 mL/min. The column was deactivated with 0.5 M ethanolamine, 0.5 M NaCl, pH 8.3 followed by 0.1 M acetate, 0.5 M NaCl, pH 4.0. Using this method, 10 mg of rGT2 was coupled to the column.

For the enrichment of GT2-specific immunoglobulins, the GT2-affinity column was equilibrated with wash buffer (50 mM phosphate buffer, 0.5 M NaCl, pH 7.0 and loaded at 1 mL/min flow rate with a 5 mL volume of sheep antisera from an animal vaccinated with rGT2. Bound anti-GT2 immunoglobulins were eluted with 0.1 M NaH_2_PO_4_, pH 3.0 and then neutralised with 1 M NH_4_HCO_3_. The GT2-specific IgG in eluant fractions was further purified using a Protein G column (GE HealthCare) according to the manufacturers instructions. The purity of the GT2-specific IgG was confirmed by SDS-PAGE and comparison to a commercial purified sheep IgG (Jackson ImmunoResearch).

### Statistical analysis

Analysis of level of total IgA and IgG in sheep serum, saliva and rumen contents was performed on log_10_-transformed data using Minitab V.16.22 (Minitab, USA). One-way ANOVA followed by a Fisher’s multiple comparisons test was performed to compare level of IgA and IgG in different samples. Analysis of total and rGT2-specific antibody levels were based on mixed effects model using package ‘nlme’ [21] in R version 3.1 [22]. The immunoglobulin stability experiment was a randomised block design with two treatments “group” and “time”, a mixed effects model with fixed effects “group”, “time” and their interaction, and a random effect “animal” was used in the analysis. For matching the assumption of normality, either square root or log_e_ transformation was applied to the response variable. For the vaccine trial, unit values of antibody responses in sheep were log_10_-transformed prior to statistical analysis. To detect groups of different adjuvants and weeks and their interactions, day 0 data (before vaccination) were treated as covariate, groups and days and their interactions as fixed effects, and individual animals as random effects. Individual comparisons were made using a post-hoc multiple comparison test [23]. The P values from the test were adjusted by the “BH” method to control for false discovery rate [24]. The level of significance was set at a P value of <0.05.

## Results

### Level of sheep IgA and IgG in serum, saliva and rumen

There were significant differences between levels of IgA and IgG in serum, saliva and rumen contents (P < 0.05; Table 2). The mean concentration of total IgA in saliva was 7-fold higher than that of IgG, while IgA in rumen contents was 12-fold higher than IgG. In contrast, the mean concentrations of IgG in serum was 131-fold higher than that of IgA.

**Table 2:** Levels of sheep IgA and IgG in serum, saliva and rumen contents from sheep (n = 30).

Sample	Total IgA	Total IgG
Serum	152 ± 17.0^a^	19,931 ±1,454^b^
Saliva	274 ± 23.7^c^	41.7 ± 3.85^d^
Rumen contents	2.34 ± 0.44^e^	0.20 ± 0.04^f^

Data are presented in as mean concentration (μg/mL) ± SE. Different letters denoted significant differences, P <0.05.

### Stability of sheep IgA and IgG in rumen content samples

**Fig 1:**
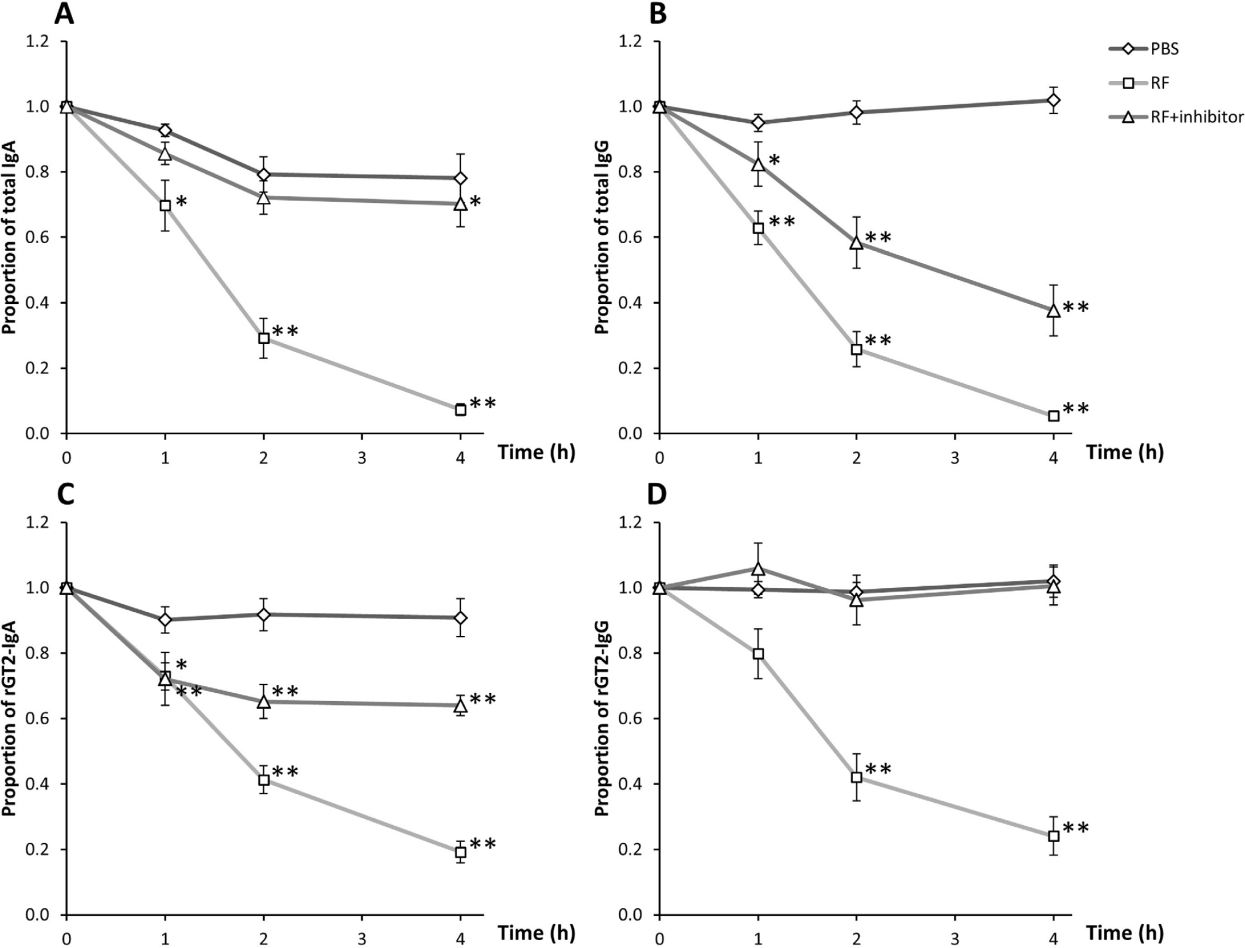
**Stability of total sheep IgA (A) and IgG (B) (n = 15), rGT2-specific IgA (C) and IgG (D) (n = 10) exposed to rumen contents from sheep.** Saliva was added to rumen contents with or without addition of protease inhibitor or added to PBS. Ig concentrations were measured by ELISA. Data are presented as mean (± SE) proportion of antibody relative to levels at time 0. Significant differences to time 0 were shown as *P < 0.05, **P < 0.001.

**Fig 2:**
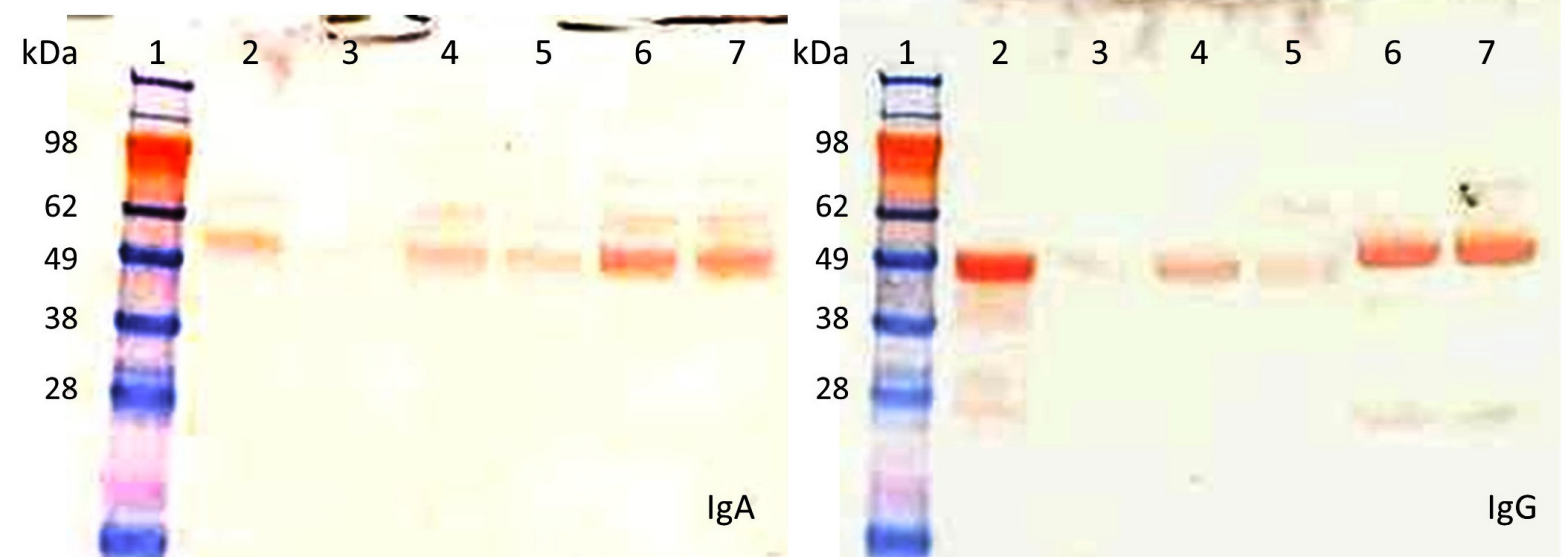
**Western blot analysis showing the stability of sheep IgA (left) and IgG (right) before (0 h) and after (4 h) incubation with sheep rumen contents or PBS at 39°C.** Lane 1: standards of different molecular weights; lane 2: rumen contents (0 h); lane 3: rumen contents (4 h); lane 4: rumen contents with protease inhibitor (0 h); lane 5: rumen contents with protease inhibitor (4 h); lane 6: PBS (0 h) and lane 7: PBS (4 h).

Fresh saliva was added to fresh rumen contents and the levels of Ig were followed over time. The levels of total and rGT2-specific IgA and IgG in rumen contents decreased by approximately 50% between 0 and 1.5 hours incubation (P < 0.001) and the levels declined even further by 4 hours (Fig 1). In contrast, when the saliva was added to PBS, the levels of total and rGT2-specific IgA and IgG remained at similar levels for up to 4 hours compared to time 0. When a protease inhibitor was added to the saliva-rumen fluid prior to incubation, the loss of detectable Ig was slowed. Total IgA decreased only slightly between 0 and 4 hours (P < 0.05). Most of the decrease of rGT2-specific IgA in the presence of protease inhibitor occurred in the first hour, with little further loss of activity from 1 to 4 hours. The level of total IgG decreased by approximately 50% between 0 and 4 hours (P < 0.001) in the presence of inhibitor whereas the levels of rGT2-specific IgG remained constant. Western blot analysis confirmed that degradation of sheep IgA and IgG had occurred when Ig was incubated in sheep rumen contents without addition of protease inhibitor, and that the presence of protease inhibitor resulted in less degradation (Fig 2).

### Antigen-specific antibody responses

**Fig 3:**
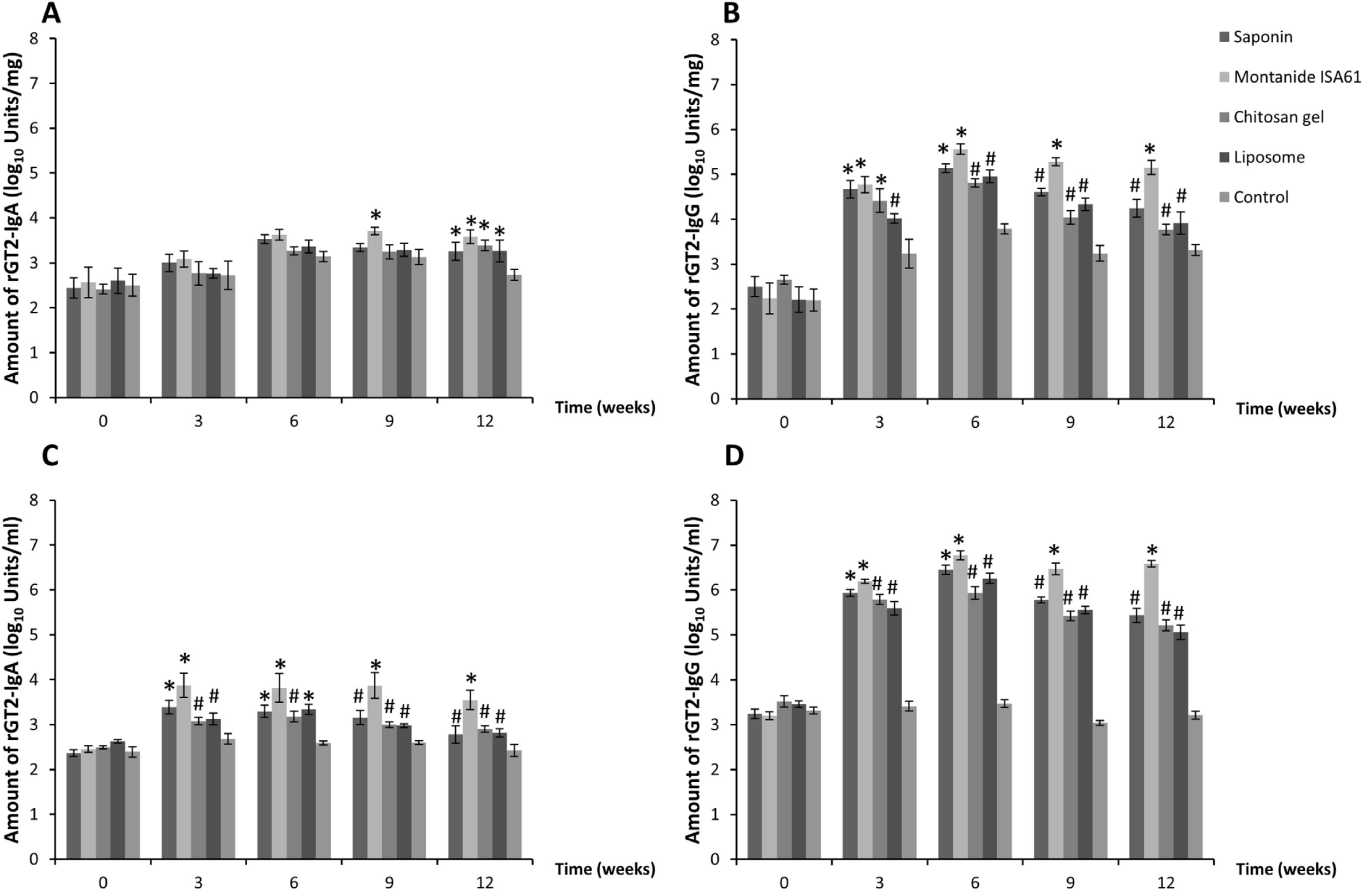
**rGT2-specific IgA & IgG in saliva (A and B, respectively) and serum (C and D, respectively) from sheep vaccinated with rGT2 antigen formulated with saponin (n = 6), Montanide ISA61 (n = 6), chitosan thermogel (n = 6), lipid nanoparticles/cationic liposomes (n = 6) and non-vaccinated control animals (n = 6).** Antibody responses were measured using ELISA. Week 0 data were treated as covariate. Saliva data were standardized by total IgA and IgG to control for different saliva flow rates between animals. Data are presented as mean log_10_ units per mg of total protein ± SE. Significant differences to non-vaccinated control animals were shown as ^#,^*P < 0.05. Different symbols denoted significant differences between groups within sampling week, P < 0.05.

Low levels of rGT2-specific IgA and IgG were detected in saliva and serum prior to vaccination (Fig 3). Compared to non-vaccinated control animals, sheep vaccinated with rGT2 formulated with the various adjuvants developed specific IgA and IgG antibody responses in saliva and serum (significant differences shown in Fig 3, P < 0.05). rGT2-specific IgA and IgG responses in saliva and serum peaked at about 6–9 weeks after the initial vaccination. The mean rGT2-specific IgA and IgG concentrations in saliva and serum of the animals vaccinated using rGT2 formulated with Montanide ISA61 (animal group 2) were the highest and were maintained at higher levels at 12 weeks compared to the other adjuvants used (P < 0.05).

Generally low levels of rGT2-specific antibody were measured in the rumen of the vaccinated animals. Elevated rGT2-specific IgA (defined as a 4-fold increase in units compared to pre-vaccination levels) were observed in some animals in group 1 (1/6 animals), group 2 (1/6 animals), group 3 (2/6 animals) and group 4 (1/6 animals) at week 6, and in group 1 (1/6 animals), group 2 (3/6 animals), group 3 (3/6 animals) and group 4 (1/6 animals) at week 12. There was an increase in rGT2-specifc IgG, in group 2 (3/6 animals), group 3 (3/6 animals) and group 4 (2/6 animals) at week 6, and in group 2 (3/6 animals) at week 12.

**Fig 4:**
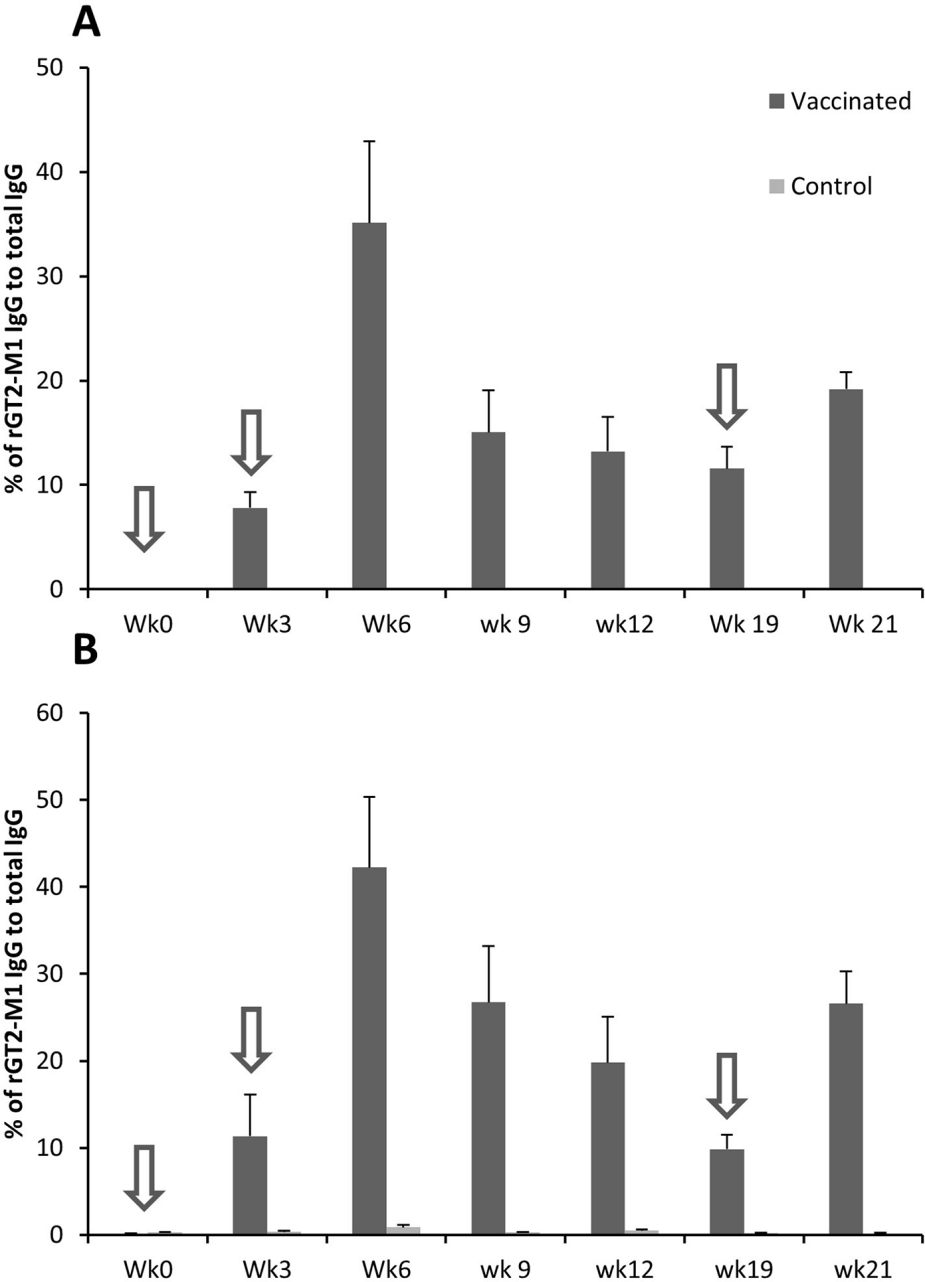
**Mean (± SE) percentage of antigen-specific IgG in serum (A) and saliva (B) of sheep vaccinated with rGT2 formulated with Montanide ISA61 (n = 6) and non-vaccinated controls (n = 6).** Arrows indicates timing of vaccinations.

Fig 4 shows the percentage of antigen-specific IgG in total IgG in serum and saliva over time after vaccination for group 2 (Montanide ISA61). Peak responses of serum and salivary IgG occurred at week 6 (3 weeks after the boosting vaccination) and then declined slowly. At week 6, antigen-specific levels of antibody in saliva were 43% of the total IgG. Considering the concentration of total IgG in saliva is 41.7 μg/mL (4.17 × 10^−2^ g/L, Table 2), we calculated that at this time point saliva would contain 1.79 × 10^−2^ g/L antigen-specific IgG. This would equate to a concentration of 7.2 × 10^16^ antigen-specific molecules of IgG/L saliva. At week 19, i.e., 16 weeks after the boosting vaccination, the antigen-specific IgG in saliva remained at 10% of the total IgG. Following a single re-vaccination at week 19, the level of antigen-specific IgG increased to 27% of the total IgG at week 21.

## Discussion

Identifying suitable antigens for a vaccine, an effective adjuvant system, and a route of vaccination for delivering substantial and continuous levels of salivary antibodies will be crucial for the success of an anti-methanogen vaccination strategy. Knowledge of antibody stability in the rumen will help in understanding the levels of antibody that need to be induced in the saliva to ensure sufficient active antibodies are present in the rumen. In this study, we confirmed the major class of immunoglobulin in salivary antibody, evaluated the stability of Ig in sheep rumen contents and identified an adjuvant (Montanide ISA61 administered subcutaneously) which induced higher levels of salivary antibody in comparison to other adjuvants. Our studies and calculations suggest that this vaccination regime can produce sufficient numbers of antigen-specific antibody molecules in the rumen to target ruminal methanogens. Collectively, the results from this study confirm the feasibility of a vaccination-based mitigation strategy for livestock generated methane.

The immunogen used to vaccinate the sheep was a fusion of the large extracellular domain from *Mbb*. *ruminantium* M1 GT2 with a commonly used tag, thioredoxin. The thioredoxin was used to aid solubility of the recombinant protein. It was not practical to cleave off the GT2 component from thioredoxin to produce enough rGT2 protein for vaccination purposes and thus the thioredoxin-GT2 fusion was used as the immunogen for our studies. It would be expected that a proportion of antibodies generated by this vaccine would be directed to thioredoxin but since GT2 is the larger fusion partner, approximately 30.5 kDa compared to a molecular size of 11.7 kDa for thioredoxin, the majority of vaccine-induced antibodies would be against the GT2 component.

The major class of immunoglobulin in saliva and the rumen of sheep was IgA. In contrast, in serum the major class was IgG. The levels of sheep serum IgG and IgA observed in this study were consistent with a previous report which found levels of IgG ranged between 17–20 mg/mL and levels of IgA ranged between 0.1–0.5 mg/mL, respectively [25]. In the current study, salivary IgA was lower than the level (0.9 mg/mL) previously reported [25], but that may have been affected by dilution caused by stimulation of saliva flow during collection, without increasing Ig production. Levels of total IgA and IgG were very low in the rumen which may be due to non-specific binding of antibody to plant material and microbes present in the rumen environment or to degradation.

Our results, using an *in vitro* assay and monitoring antibody integrity by ELISA and Western blotting, suggest that Ig is relatively stable in the rumen, with both IgA and IgG classes of immunoglobulin in saliva having a half-life of approximately 1.5–2 hours. In a previous study, it was suggested that antibodies can persist for up to 8 hours in the rumen [26]. We have recently shown that antibody, particularly IgA is relatively stable in cattle rumen contents, retaining 60% of binding in an ELISA based assay after 8 hours exposure to rumen contents [27]. The current study in sheep suggests that approximately 50% of antibodies will still be active 1–2 hours after entering the rumen. Thus it is likely that vaccine-induced antibodies will be sufficiently robust to survive in the rumen long enough to interact with the intended targets on methanogen cells. As was the case with bovine antibody [27], degradation of antibody appeared to be mediated mainly by proteases, as inclusion of a broad spectrum protease inhibitor to the assay system reduced loss of antibody binding. Interestingly, addition of protease inhibitor only partially blocked loss of total IgG binding, which may indicate that some loss of total IgG during the time course of the assay was mediated via a non-protease degradation mechanism. In contrast, loss of antigen-specific IgG was completely abrogated by addition of protease inhibitor, suggesting there was minimal non-specific binding of antigen-specific antibody to material in the rumen contents. Since the rumen contents were collected from rGT2-vaccinated sheep, it is possible that the majority of antibody-binding sites in rumen material, either microbial or plant-derived were already blocked by saliva-derived antibody.

Antibody was detected in rumen samples from only a proportion of the vaccinated animals, and levels were considerably lower than in the saliva. The low levels of antigen-specific antibody measured in the rumen could be due to binding of antibody to native GT2 protein on the surface of methanogens. *Mbb*. *ruminantium* M1 is present at only low abundance in the rumen [28] but it is possible that antibody may have cross-reacted with other methanogens within the *Mbb*. *ruminantium* clade. In addition, non-specific binding of antibody to microbes, plant material, or to other matter in the rumen may have occurred. Degradation of specific antibody by proteases in the rumen could also account for some loss of antibody as our results suggest that a proportion of antibody is lost due to proteolytic degradation.

Montanide ISA61 is a commercially-available water-in-oil adjuvant that has previously been shown to promote strong antibody responses to a model antigen, tetanus toxoid [11]. In the current study, a methanogen antigen formulated with Montanide ISA61 and given subcutaneously induced primarily IgG with only moderate levels of IgA. Vaccination induced antigen-specific antibodies in the saliva and these antibodies were also delivered to the rumen. Formulation of rGT2 with Montanide ISA61 produced 5-fold higher levels of rGT2-specific salivary IgG compared to the levels of antibody produced using the second best adjuvant, saponin.

The amount of antigen-specific antibody required to be delivered to rumen for a vaccine to be effective to reduce methane emissions is presently unknown and it would be useful to understand the level of antigen-specific antibodies required to inhibit methanogens in the rumen. The titres of enhanced antigen-specific IgG in saliva and serum reported in the current study were consistent with previous reports of effective vaccination of sheep against bacteria causing lactic acidosis. Those studies reported a 2 and 3-log increase in antigen-specific total Ig in saliva and serum, respectively and these levels were protective against lactic acidosis in sheep [4, 6]. The levels of IgG in sera and saliva in the current study are also similar to those reported for vaccination of dairy cows against bacterial urease [10]. In order to better understand the amount of methanogen antigen-specific antibody being generated in the saliva and delivered to the rumen, an anti-rGT2 IgG standard was prepared and used to calculate percentage of the total IgG and number of antigen-specific IgG molecules generated in sera and saliva. The percentage of antigen-specific IgG in serum and saliva were similar, suggesting that the rGT2-specific IgG in the saliva of the vaccinated sheep were derived from serum. In addition, it has been shown that the level of antigen-specific antibody in serum and saliva remained at approximately 10% of total IgG after 4 months and this can be increased by a single boosting vaccination. Our calculations suggested that vaccination of sheep with rGT2 formulated with Montanide ISA61 stimulated up to 7 × 10^16^ molecules of target-specific IgG per litre of saliva. Assuming that saliva input into the rumen is about the same as the rumen liquid outflow [29], and that there are approximately 10^9^ methanogens per millilitre of rumen contents, then the current vaccination strategy would result in an input specific IgG that would keep the ruminal concentration at approximately 72,000 molecules of antigen-specific antibody for each methanogen cell. The majority of these antibodies are likely to be available for binding to immunogenic epitopes of GT2 proteins located on the surface of ruminal methanogens, as our results suggested only slow loss of antibody activity (half-life of approximately 1.5 h) in rumen contents. While antigen-specific antibody levels peaked three weeks after the boosting vaccination, at 16 weeks post boosting there would still be greater than 16,000 molecules potentially available for binding to each methanogen cell in the rumen. Saliva is continually produced in large quantities by ruminants, and once antibodies to selected methanogen antigenic targets have been generated in saliva via vaccination, the rumen would be constantly replenished with fresh antibody potentially available for binding to methanogens.

In conclusion, our results confirmed that sheep vaccinated with a model methanogen antigen produced antigen-specific antibody in both serum and saliva and showed levels of salivary antibody are influenced by vaccine formulation and adjuvant. While antibody can remain relatively stable in the rumen for 1–2 hours, an effective vaccination strategy will need to use potent adjuvants to formulate the antigens to ensure sufficiently high levels of antibody are available in the rumen for interaction with their intended targets on the surface of methanogens. Our results suggest that formulation of a methanogen vaccine antigen with Montanide ISA61 adjuvant and given to sheep via a parenteral route can generate theoretically high enough levels of antigen-specific antibody in the saliva to effectively target ruminal methanogens. The ultimate success of a vaccination approach for reducing methane production in the rumen will depend on selecting targets on the surface of methanogen cells which are both critical for cell function and accessible for antibody binding.
